# Supplementary material for: A new stress field intensity model and its application in component high cycle fatigue research
Source: PLoS One. 2020 Jul 21;15(7):e0235323. doi: 10.1371/journal.pone.0235323 (PMC7373292; doi:10.1371/journal.pone.0235323)
Supplement: S1 File — (DOCX) [file pone.0235323.s001.docx]

| Nomenclature  $\sigma_{FI}$- stress field intensity  $V$- damage volume  $\Omega$- stress field range  $f(\sigma_{ijk})$- stress damage function (usually Von mises stress)  - weight function $\sigma_{b}$  - fatigue limit of the material  $\sigma_{FE}$, - stress obtained by the finite element approach  $\sigma_{i}$- stress obtained by the distribution function  $f$- relative difference between the distribution function and the finite element results  $M_{e}$- prediction of the fatigue limit load of a new crankshaft  $\sigma_{FI}(A)$- the stress field intensity of the second crankshaft under a bending moment of 1000 N∙m |
| --- |
