# Supplementary material for: A new stress field intensity model and its application in component high cycle fatigue research
Source: PLoS One. 2020 Jul 21;15(7):e0235323. doi: 10.1371/journal.pone.0235323 (PMC7373292; doi:10.1371/journal.pone.0235323)
Supplement: Attachment — Submitted filename: Response to Reviewers.docx [file pone.0235323.s002.docx]

Dear Editors:

On behalf of my co-authors, I am submitting the revised manuscript “A new stress field intensity model and its application in component high cycle fatigue research” for possible publication in Plos one.

In last month, I submitted this manuscript to the journal. Soon afterwards it was sent back for major revision. We have studied reviewers’ comments carefully. According to the reviewers’ detailed suggestions, we have made a careful revision on the original manuscript. All revised portions are marked in red in the revised manuscript which we would like to submit for your kind consideration.

Replies to the reviewers’ comments:

Reviewer #1:

1. For example, what does “field diameter” mean?

Response：the definition of field diameter has been introduced in the introduction part (under equation 1.4)

2. I would’t call the loads applied on the crankshaft as “impacts” because it is not the case, otherwise you should employ a completely different structural integrity approach.

Response: the load has been renamed as dynamic load

3. On top of that, it is ignored completely the multiaxiality issue of this case-study.

Response: the stress field intensity approach is just considered to be an effective tool in researching the multiaxial fatigue problems, we have explained in the revised manuscript.

Reviewer #2:

1. This topic was previously studied by the author, Ref. # 1. Therefore, the author should explain the novelty in this manuscript.

Response: the novelty in this manuscript is the new modified stress field model, we have explained in the manuscript.

2. Some previous important contributions in the literature are not included in both introduction and discussion, See for example the following Refs.:

a. "Significance of crack tip plasticity to early notch fatigue crack growth", Int. J. of Fatigue, Vol. 26, No. 2, pp. 173-182, 2004.

b. "Mode I notch fatigue crack growth behaviour under constant amplitude loading and due to the application of a single tensile overload", Int. J. of Fatigue, Vol. 26, No. 2, pp. 183-192, 2004.

c. "Deformation behaviour at the tip of physically short fatigue crack due to a single overload", Fatigue Fract. Engng Mater. Struct., Vol. 22,145-151, 1999.

Response: these articles have been added to the reference.

3. The English language should be polished throughout the manuscript

Response: the whole manuscript has been edited by the AJE company.

4. Abstract should be extended

Response: the abstract has been extended

Reviewer #3:

1. In introduction section, the author introduces the stress field intensity method and compares it with the TCD method. The author should address more details about this stress field intensity method. For example, how does it work, why does this paper choose this method other than TCD which is similar to the stress field intensity method?

Response: the introduction of the stress field intensity approach has been removed to the introduction section, and the reason of the choice has been explained (the feasibility in researching the multiaxial fatigue problems)

2. In section 2.2. The Eq. (2.5) is used as an assumed stress distributed function. The author should provide details about this assumption. If this assumption comes from other studies, the reference should be provided. If this equation is assumed by the author, more details and the limitation of this assumption should be provided.

Response: the distribution function comes from Adrea’s article, which has been added in the manuscript.

3. In Section 3.1, the author gives the test results (Tab. 1) and the corresponding finite element model (Fig. 2). The model details should be provided, such as material properties, temperature, boundary condition, and model dimensions. Without this data, such experiments and simulations cannot be repeated by others.

Response: the detailed information of the finite element model has been added in the manuscript, combined with the structural parameters in Tab.13, the simulation can be repeated.

4. What is the difference between Case one and Case two?

Response: the difference is the material, which has been stated by the S-N curves in Fig.5 and Fig.8.

Reviewer #4:

1. The Introduction needs to be significantly improved. There are certain research groups (Marsavina et al., Linul et al., Berto et al., Sadowski et al., etc.) dealing with the fatigue property prediction and not only. Please refer to their works in the Introduction.

Response: these works have been added in the introduction part.

2. The standard deviations should be added in the Tables.

Response: we are sorry but we can’t understand which tables should be added with the standard deviations, because the parameters in the tables are just finite element analysis results, structural parameters, and experiment results. They are not statistical data. Please indicate this more clearly

3. The error bars should be added in the Figures 4-7.

Response: the errors of the fitting results are too small to be expressed by curves, so we added four more tables(4,5,8,9) to explain them more clearly

4. It is recommended to draw Wöhler curves.

Response: the curves have been added in both cases.

5. The used standard must be presented.

Response: we have added the experiment standard in the manuscript.

6. References must be uniformized. Some journals are abbreviated and others are not.

Response: the references have been uniformized.

7. The literature review should consider more in details the recent developments published in the PLOS ONE journal, showing a continuity between the present paper and those reported in the literature on similar topics.

Response: some articles have been added(ref.8&12)

8. English is not the native language of this Reviewer; however, there are a number of spelling errors and other mistakes in grammar. Therefore, proof-reading of the paper for correct English would be highly advisable.

Response: the whole manuscript has been edited by the AJE company.

Best Regards.

Yours Sincerely

Sun Songsong

E-mail:sunsong1987@126.com
